# 3-(4-Chloro­phenyl­sulfin­yl)-2,4,7-tri­meth­yl-1-benzo­furan

**DOI:** 10.1107/S160053681302117X

**Published:** 2013-08-03

**Authors:** Hong Dae Choi, Pil Ja Seo, Uk Lee

**Affiliations:** a Department of Chemistry, Dongeui University, San 24 Kaya-dong, Busanjin-gu, Busan 614-714, Republic of Korea; b Department of Chemistry, Pukyong National University, 599-1 Daeyeon 3-dong, Nam-gu, Busan 608-737, Republic of Korea

## Abstract

In the title compound, C_17_H_15_ClO_2_S, the dihedral angle between the mean plane [r.m.s. deviation = 0.020 (2) Å] of the benzo­furan ring system and the mean plane [r.m.s. deviation = 0.011 (1) Å] of the 4-chloro­phenyl ring is 72.68 (6)°. In the crystal, mol­ecules are linked *via* pairs of C—H⋯π inter­actions into inversion dimers. These dimers are further packed by C—H⋯O hydrogen bonds into supra­molecular chains running along the *a*-axis direction. In addition, the crystal structure also exhibits π–π inter­actions between the 4-chloro­phenyl rings of adjacent mol­ecules [centroid–centroid distance = 4.094 (3) Å, inter­planar distance = 3.648 (3) Å and slippage = 1.656 (3) Å].

## Related literature

For background information and the crystal structures of related compounds, see: Choi *et al.* (2010*a* ▶,*b* ▶). 
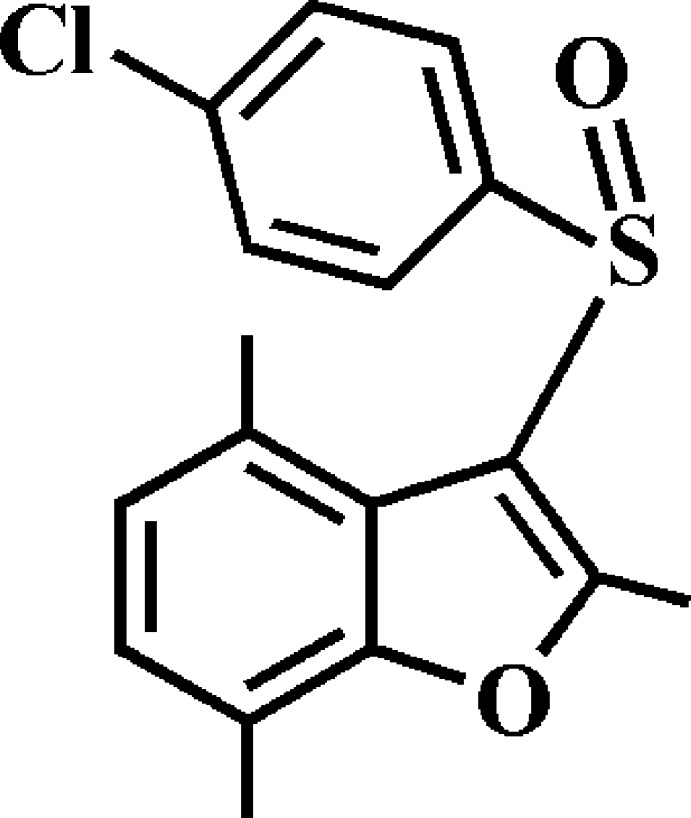


## Experimental

### 

#### Crystal data

C_17_H_15_ClO_2_S
*M* *_r_* = 318.80
Triclinic, 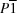

*a* = 6.043 (2) Å
*b* = 11.716 (4) Å
*c* = 12.217 (5) Å
α = 117.99 (2)°
β = 92.00 (2)°
γ = 97.42 (2)°
*V* = 752.9 (5) Å^3^
*Z* = 2
Mo *K*α radiation
μ = 0.39 mm^−1^
*T* = 173 K
0.31 × 0.24 × 0.17 mm

#### Data collection

Bruker SMART APEXII CCD diffractometer
Absorption correction: multi-scan (*SADABS*; Bruker, 2009 ▶) *T*_min_ = 0.633, *T*_max_ = 0.746
12137 measured reflections
3192 independent reflections
2515 reflections with *I* > 2σ(*I*)
*R*_int_ = 0.036

#### Refinement

*R*[*F* ^2^ > 2σ(*F* ^2^)] = 0.040
*wR*(*F* ^2^) = 0.128
*S* = 1.12
3192 reflections
193 parameters
H-atom parameters constrained
Δρ_max_ = 0.48 e Å^−3^
Δρ_min_ = −0.55 e Å^−3^

### 

Data collection: *APEX2* (Bruker, 2009 ▶); cell refinement: *SAINT* (Bruker, 2009 ▶); data reduction: *SAINT*; program(s) used to solve structure: *SHELXS97* (Sheldrick, 2008 ▶); program(s) used to refine structure: *SHELXL97* (Sheldrick, 2008 ▶); molecular graphics: *ORTEP-3* for Windows (Farrugia, 2012 ▶) and *DIAMOND* (Brandenburg, 1998 ▶); software used to prepare material for publication: *SHELXL97*.

## Supplementary Material

Crystal structure: contains datablock(s) I. DOI: 10.1107/S160053681302117X/pk2491sup1.cif

Structure factors: contains datablock(s) I. DOI: 10.1107/S160053681302117X/pk2491Isup2.hkl

Supplementary material file. DOI: 10.1107/S160053681302117X/pk2491Isup3.cml

Additional supplementary materials:  crystallographic information; 3D view; checkCIF report

## Figures and Tables

**Table 1:** Hydrogen-bond geometry (Å, °) *Cg*1 is the centroid of the C1/C2/C7/O1/C8 furan ring.

*D*—H⋯*A*	*D*—H	H⋯*A*	*D*⋯*A*	*D*—H⋯*A*
C11—H11*A*⋯O2^i^	0.98	2.38	3.254 (3)	148
C11—H11*B*⋯*Cg*1^ii^	0.98	2.96	3.503 (3)	116

Symmetry codes: (i) 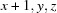; (ii) 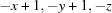.

**Fig. 1.**
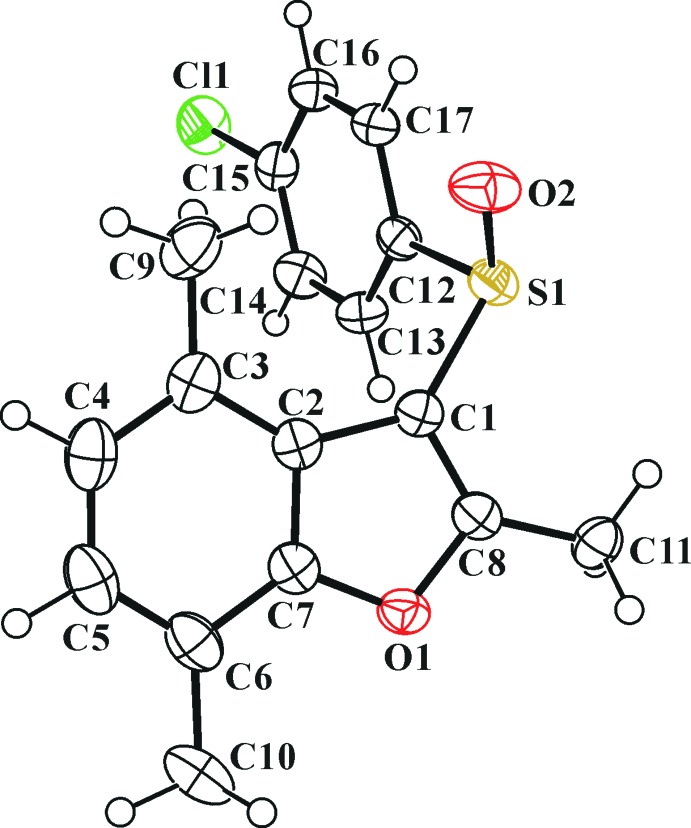
The molecular structure of the title compound. Displacement ellipsoids are drawn at the 50% probability level. H atoms are presented as small spheres of arbitrary radius.

**Fig. 2.**
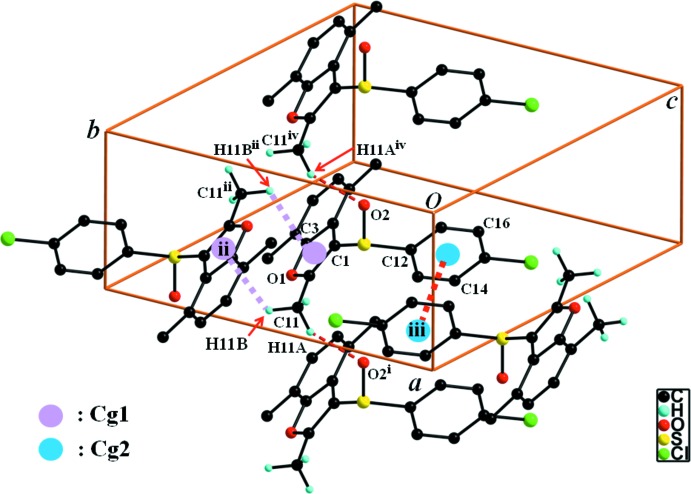
A view of the C—H···O, C—H···π and π–π interactions (dotted lines) in the crystal structure of the title compound. H atoms not participating in hydrogen-bonding were omitted for clarity. [Symmetry codes: (i) x + 1, y, z; (ii) - x + 1, - y + 1, - z; (iii) - x + 1, - y, - z; (iv) x - 1, y, z.]

## supplementary crystallographic information

### 1. Comment

As a part of our ongoing study of 2,5,7-trimethyl-1-benzofuran derivatives containing 4-fluorophenylsulfinyl (Choi *et al.*, 2010*a*) and 4-chlorophenylsulfinyl (Choi *et al.*, 2010*b*) substituents in 3-postion, we report herein the crystal structure of the title compound.

In the title molecule (Fig. 1), the benzofuran unit is essentially planar, with a mean deviation of 0.020 (2) Å from the least-squares plane defined by the nine constituent atoms. The 4-chlorophenyl ring is essentially planar, with a mean deviation of 0.011 (1) Å from the least-squares plane defined by the six constituent atoms. The dihedral angle formed by the benzofuran ring system and the 4-chlorophenyl ring is 72.68 (6)°. In the crystal structure (Fig. 2), molecules are connected via pairs of C—H···π interactions into inversion dimers (Table 1, Cg1 is the centroid of the C1/C2/C7/O1/C8 furan ring). These dimers are further packed by C—H···O hydrogen bonds (Table 1) into supramolecular chains running along the *a*-axis direction. Additionally, the crystal packing (Fig. 2) also exhibits π–π interactions between the 4-chlorophenyl rings of adjacent molecules, with a Cg2···Cg2^iii^ distance of 4.094 (3) Å and an interplanar distance of 3.648 (3) Å resulting in a slippage of 1.656 (3) Å (Cg2 is the centroid of C12–C17 4-chlorophenyl ring).

### 2. Experimental

3-Chloroperoxybenzoic acid (77%, 269 mg, 1.2 mmol) was added in small portions to a stirred solution of 3-(4-chlorophenylsulfanyl)-2,4,7-trimethyl-1-benzofuran (333 mg, 1.1 mmol) in dichloromethane (40 mL) at 273 K. After being stirred at room temperature for 4h, the mixture was washed with saturated sodium bicarbonate solution and the organic layer was separated, dried over magnesium sulfate, filtered and concentrated at reduced pressure. The residue was purified by column chromatography (hexane-ethyl acetate, 4:1 v/v) to afford the title compound as a colorless solid [yield 66%, m.p. 432–433 K; *R*_f_ = 0.45 (hexane-ethyl acetate, 4:1 v/v)]. Single crystals suitable for X-ray diffraction were prepared by slow evaporation of a solution of the title compound in ethyl acetate at room temperature.

### 3. Refinement

All H atoms were positioned geometrically and refined using a riding model, with C—H = 0.95 Å for aryl and 0.99 Å for methyl H atoms, respectively. *U*_iso_(H) = 1.2*U*_eq_(C) for aryl and 1.5*U*_eq_(C) for methyl H atoms. The positions of methyl hydrogens were optimized rotationally.

### Crystal data

**Table:**

C_17_H_15_ClO_2_S	*Z* = 2
*M**_r_* = 318.80	*F*(000) = 332
Triclinic, *P*1	*D*_x_ = 1.406 Mg m^−^^3^
Hall symbol: -P 1	Melting point = 432–433 K
*a* = 6.043 (2) Å	Mo *K*α radiation, λ = 0.71073 Å
*b* = 11.716 (4) Å	Cell parameters from 4880 reflections
*c* = 12.217 (5) Å	θ = 3.3–28.5°
α = 117.99 (2)°	µ = 0.39 mm^−^^1^
β = 92.00 (2)°	*T* = 173 K
γ = 97.42 (2)°	Block, colourless
*V* = 752.9 (5) Å^3^	0.31 × 0.24 × 0.17 mm

### Data collection

**Table:**

Bruker SMART APEXII CCD diffractometer	3192 independent reflections
Radiation source: rotating anode	2515 reflections with *I* > 2σ(*I*)
Graphite multilayer monochromator	*R*_int_ = 0.036
Detector resolution: 10.0 pixels mm^-1^	θ_max_ = 27.0°, θ_min_ = 1.9°
φ and ω scans	*h* = −7→7
Absorption correction: multi-scan (*SADABS*; Bruker, 2009)	*k* = −14→14
*T*_min_ = 0.633, *T*_max_ = 0.746	*l* = −15→15
12137 measured reflections	

### Refinement

**Table:**

Refinement on *F*^2^	Primary atom site location: structure-invariant direct methods
Least-squares matrix: full	Secondary atom site location: difference Fourier map
*R*[*F*^2^ > 2σ(*F*^2^)] = 0.040	Hydrogen site location: difference Fourier map
*wR*(*F*^2^) = 0.128	H-atom parameters constrained
*S* = 1.12	*w* = 1/[σ^2^(*F*_o_^2^) + (0.0707*P*)^2^ + 0.168*P*] where *P* = (*F*_o_^2^ + 2*F*_c_^2^)/3
3192 reflections	(Δ/σ)_max_ < 0.001
193 parameters	Δρ_max_ = 0.48 e Å^−^^3^
0 restraints	Δρ_min_ = −0.55 e Å^−^^3^

### Special details

**Table:**

Geometry. All esds (except the esd in the dihedral angle between two l.s. planes) are estimated using the full covariance matrix. The cell esds are taken into account individually in the estimation of esds in distances, angles and torsion angles; correlations between esds in cell parameters are only used when they are defined by crystal symmetry. An approximate (isotropic) treatment of cell esds is used for estimating esds involving l.s. planes.
Refinement. Refinement of F^2^ against ALL reflections. The weighted R-factor wR and goodness of fit S are based on F^2^, conventional R-factors R are based on F, with F set to zero for negative F^2^. The threshold expression of F^2^ > 2sigma(F^2^) is used only for calculating R-factors(gt) etc. and is not relevant to the choice of reflections for refinement. R-factors based on F^2^ are statistically about twice as large as those based on F, and R- factors based on ALL data will be even larger.

### Fractional atomic coordinates and isotropic or equivalent isotropic displacement parameters (Å^2^)

**Table:**

	*x*	*y*	*z*	*U*_iso_*/*U*_eq_	
Cl1	0.57368 (11)	−0.03761 (6)	0.34385 (5)	0.0487 (2)	
S1	0.33471 (8)	0.22715 (5)	0.02081 (5)	0.03169 (17)	
O1	0.7881 (2)	0.53758 (12)	0.14079 (13)	0.0322 (3)	
O2	0.0894 (2)	0.22965 (15)	0.02338 (16)	0.0456 (4)	
C1	0.4952 (3)	0.38358 (17)	0.10912 (18)	0.0276 (4)	
C2	0.5111 (3)	0.48591 (18)	0.23757 (18)	0.0294 (4)	
C3	0.3881 (4)	0.5125 (2)	0.3376 (2)	0.0366 (5)	
C4	0.4708 (5)	0.6281 (2)	0.4462 (2)	0.0471 (6)	
H4	0.3926	0.6495	0.5176	0.057*	
C5	0.6598 (4)	0.7135 (2)	0.4561 (2)	0.0475 (6)	
H5	0.7066	0.7902	0.5337	0.057*	
C6	0.7820 (4)	0.69163 (19)	0.3584 (2)	0.0392 (5)	
C7	0.6962 (3)	0.57630 (18)	0.25069 (19)	0.0313 (4)	
C8	0.6624 (3)	0.42057 (17)	0.05637 (18)	0.0290 (4)	
C9	0.1778 (4)	0.4253 (2)	0.3290 (2)	0.0486 (6)	
H9A	0.0852	0.3988	0.2509	0.073*	
H9B	0.0943	0.4726	0.3997	0.073*	
H9C	0.2157	0.3474	0.3308	0.073*	
C10	0.9885 (4)	0.7807 (2)	0.3643 (2)	0.0527 (7)	
H10A	1.0121	0.8601	0.4455	0.079*	
H10B	0.9702	0.8046	0.2980	0.079*	
H10C	1.1185	0.7357	0.3530	0.079*	
C11	0.7331 (4)	0.36120 (19)	−0.06862 (19)	0.0343 (5)	
H11A	0.8690	0.3239	−0.0681	0.051*	
H11B	0.7646	0.4281	−0.0952	0.051*	
H11C	0.6132	0.2917	−0.1266	0.051*	
C12	0.4109 (3)	0.15774 (17)	0.11811 (17)	0.0270 (4)	
C13	0.6311 (3)	0.17380 (19)	0.1672 (2)	0.0323 (4)	
H13	0.7474	0.2259	0.1523	0.039*	
C14	0.6813 (3)	0.1143 (2)	0.2374 (2)	0.0355 (5)	
H14	0.8307	0.1269	0.2728	0.043*	
C15	0.5108 (4)	0.03655 (18)	0.25513 (18)	0.0319 (5)	
C16	0.2927 (4)	0.01659 (19)	0.20341 (19)	0.0339 (5)	
H16	0.1778	−0.0391	0.2147	0.041*	
C17	0.2428 (3)	0.07844 (19)	0.13485 (18)	0.0312 (4)	
H17	0.0932	0.0660	0.0997	0.037*	

### Atomic displacement parameters (Å^2^)

**Table:**

	*U*^11^	*U*^22^	*U*^33^	*U*^12^	*U*^13^	*U*^23^
Cl1	0.0644 (4)	0.0447 (3)	0.0454 (3)	0.0075 (3)	−0.0040 (3)	0.0294 (3)
S1	0.0320 (3)	0.0291 (3)	0.0334 (3)	−0.00004 (19)	−0.0040 (2)	0.0162 (2)
O1	0.0341 (8)	0.0262 (7)	0.0362 (8)	0.0016 (5)	0.0023 (6)	0.0159 (6)
O2	0.0290 (8)	0.0526 (9)	0.0651 (11)	−0.0005 (7)	−0.0084 (7)	0.0389 (9)
C1	0.0292 (10)	0.0255 (9)	0.0304 (10)	0.0061 (7)	0.0021 (8)	0.0151 (8)
C2	0.0321 (10)	0.0272 (9)	0.0318 (10)	0.0088 (8)	0.0022 (8)	0.0158 (8)
C3	0.0411 (12)	0.0387 (11)	0.0367 (11)	0.0166 (9)	0.0097 (9)	0.0207 (10)
C4	0.0590 (15)	0.0478 (13)	0.0360 (12)	0.0222 (12)	0.0119 (11)	0.0174 (11)
C5	0.0614 (16)	0.0332 (11)	0.0367 (13)	0.0129 (11)	−0.0026 (11)	0.0068 (10)
C6	0.0451 (12)	0.0273 (10)	0.0405 (12)	0.0083 (9)	−0.0046 (10)	0.0124 (9)
C7	0.0353 (11)	0.0271 (9)	0.0337 (11)	0.0086 (8)	0.0009 (9)	0.0156 (8)
C8	0.0317 (10)	0.0249 (9)	0.0330 (10)	0.0050 (7)	−0.0008 (8)	0.0162 (8)
C9	0.0490 (15)	0.0514 (14)	0.0506 (14)	0.0151 (11)	0.0204 (12)	0.0260 (12)
C10	0.0501 (15)	0.0303 (11)	0.0598 (16)	−0.0017 (10)	−0.0098 (12)	0.0101 (11)
C11	0.0395 (11)	0.0349 (10)	0.0353 (11)	0.0093 (9)	0.0076 (9)	0.0212 (9)
C12	0.0284 (10)	0.0221 (8)	0.0277 (10)	0.0048 (7)	0.0019 (8)	0.0095 (8)
C13	0.0253 (10)	0.0326 (10)	0.0403 (11)	0.0033 (8)	0.0054 (9)	0.0188 (9)
C14	0.0291 (10)	0.0356 (10)	0.0423 (12)	0.0076 (8)	−0.0007 (9)	0.0187 (9)
C15	0.0415 (12)	0.0267 (9)	0.0291 (10)	0.0089 (8)	0.0056 (9)	0.0137 (8)
C16	0.0350 (11)	0.0300 (10)	0.0351 (11)	−0.0003 (8)	0.0047 (9)	0.0156 (9)
C17	0.0273 (10)	0.0309 (10)	0.0331 (11)	0.0001 (8)	0.0014 (8)	0.0146 (9)

### Geometric parameters (Å, º)

**Table:**

Cl1—C15	1.736 (2)	C9—H9A	0.9800
S1—O2	1.4876 (16)	C9—H9B	0.9800
S1—C1	1.754 (2)	C9—H9C	0.9800
S1—C12	1.803 (2)	C10—H10A	0.9800
O1—C7	1.365 (3)	C10—H10B	0.9800
O1—C8	1.367 (2)	C10—H10C	0.9800
C1—C8	1.348 (3)	C11—H11A	0.9800
C1—C2	1.450 (3)	C11—H11B	0.9800
C2—C3	1.382 (3)	C11—H11C	0.9800
C2—C7	1.386 (3)	C12—C17	1.373 (3)
C3—C4	1.392 (3)	C12—C13	1.393 (3)
C3—C9	1.491 (3)	C13—C14	1.383 (3)
C4—C5	1.379 (4)	C13—H13	0.9500
C4—H4	0.9500	C14—C15	1.377 (3)
C5—C6	1.363 (4)	C14—H14	0.9500
C5—H5	0.9500	C15—C16	1.383 (3)
C6—C7	1.389 (3)	C16—C17	1.388 (3)
C6—C10	1.497 (3)	C16—H16	0.9500
C8—C11	1.457 (3)	C17—H17	0.9500
			
O2—S1—C1	112.31 (9)	H9A—C9—H9C	109.5
O2—S1—C12	106.67 (9)	H9B—C9—H9C	109.5
C1—S1—C12	97.93 (9)	C6—C10—H10A	109.5
C7—O1—C8	106.81 (16)	C6—C10—H10B	109.5
C8—C1—C2	106.85 (17)	H10A—C10—H10B	109.5
C8—C1—S1	117.84 (15)	C6—C10—H10C	109.5
C2—C1—S1	134.94 (16)	H10A—C10—H10C	109.5
C3—C2—C7	118.84 (19)	H10B—C10—H10C	109.5
C3—C2—C1	136.4 (2)	C8—C11—H11A	109.5
C7—C2—C1	104.69 (18)	C8—C11—H11B	109.5
C2—C3—C4	115.3 (2)	H11A—C11—H11B	109.5
C2—C3—C9	122.4 (2)	C8—C11—H11C	109.5
C4—C3—C9	122.2 (2)	H11A—C11—H11C	109.5
C5—C4—C3	123.8 (2)	H11B—C11—H11C	109.5
C5—C4—H4	118.1	C17—C12—C13	120.27 (18)
C3—C4—H4	118.1	C17—C12—S1	116.75 (15)
C6—C5—C4	122.3 (2)	C13—C12—S1	122.80 (16)
C6—C5—H5	118.9	C14—C13—C12	120.40 (19)
C4—C5—H5	118.9	C14—C13—H13	119.8
C5—C6—C7	113.1 (2)	C12—C13—H13	119.8
C5—C6—C10	124.5 (2)	C15—C14—C13	118.78 (19)
C7—C6—C10	122.4 (2)	C15—C14—H14	120.6
O1—C7—C2	110.62 (17)	C13—C14—H14	120.6
O1—C7—C6	122.9 (2)	C14—C15—C16	121.26 (19)
C2—C7—C6	126.5 (2)	C14—C15—Cl1	118.78 (16)
C1—C8—O1	111.01 (18)	C16—C15—Cl1	119.97 (17)
C1—C8—C11	133.18 (18)	C15—C16—C17	119.65 (19)
O1—C8—C11	115.80 (18)	C15—C16—H16	120.2
C3—C9—H9A	109.5	C17—C16—H16	120.2
C3—C9—H9B	109.5	C12—C17—C16	119.60 (18)
H9A—C9—H9B	109.5	C12—C17—H17	120.2
C3—C9—H9C	109.5	C16—C17—H17	120.2
			
O2—S1—C1—C8	−132.20 (15)	C10—C6—C7—O1	−2.5 (3)
C12—S1—C1—C8	116.06 (16)	C5—C6—C7—C2	−2.0 (3)
O2—S1—C1—C2	55.9 (2)	C10—C6—C7—C2	177.73 (19)
C12—S1—C1—C2	−55.8 (2)	C2—C1—C8—O1	1.1 (2)
C8—C1—C2—C3	175.4 (2)	S1—C1—C8—O1	−172.94 (12)
S1—C1—C2—C3	−12.0 (4)	C2—C1—C8—C11	−179.51 (19)
C8—C1—C2—C7	−1.4 (2)	S1—C1—C8—C11	6.5 (3)
S1—C1—C2—C7	171.16 (16)	C7—O1—C8—C1	−0.4 (2)
C7—C2—C3—C4	−2.7 (3)	C7—O1—C8—C11	−179.88 (15)
C1—C2—C3—C4	−179.2 (2)	O2—S1—C12—C17	23.92 (17)
C7—C2—C3—C9	175.67 (19)	C1—S1—C12—C17	140.15 (16)
C1—C2—C3—C9	−0.8 (4)	O2—S1—C12—C13	−160.93 (16)
C2—C3—C4—C5	0.9 (3)	C1—S1—C12—C13	−44.70 (18)
C9—C3—C4—C5	−177.5 (2)	C17—C12—C13—C14	−2.6 (3)
C3—C4—C5—C6	0.5 (4)	S1—C12—C13—C14	−177.56 (15)
C4—C5—C6—C7	−0.1 (3)	C12—C13—C14—C15	1.6 (3)
C4—C5—C6—C10	−179.8 (2)	C13—C14—C15—C16	0.5 (3)
C8—O1—C7—C2	−0.6 (2)	C13—C14—C15—Cl1	−179.75 (15)
C8—O1—C7—C6	179.63 (17)	C14—C15—C16—C17	−1.6 (3)
C3—C2—C7—O1	−176.29 (16)	Cl1—C15—C16—C17	178.62 (15)
C1—C2—C7—O1	1.2 (2)	C13—C12—C17—C16	1.4 (3)
C3—C2—C7—C6	3.5 (3)	S1—C12—C17—C16	176.70 (15)
C1—C2—C7—C6	−179.02 (18)	C15—C16—C17—C12	0.6 (3)
C5—C6—C7—O1	177.81 (18)		

### Hydrogen-bond geometry (Å, º)

Cg1 is the centroid of the C1/C2/C7/O1/C8 furan ring.

**Table:**

*D*—H···*A*	*D*—H	H···*A*	*D*···*A*	*D*—H···*A*
C11—H11*A*···O2^i^	0.98	2.38	3.254 (3)	148
C11—H11*B*···*Cg*1^ii^	0.98	2.96	3.503 (3)	116

Symmetry codes: (i) *x*+1, *y*, *z*; (ii) −*x*+1, −*y*+1, −*z*.
